# Distance education for tobacco reduction with Inuit frontline health workers

**DOI:** 10.3402/ijch.v72i0.21078

**Published:** 2013-08-05

**Authors:** Rob Collins, Merryl Hammond, Catherine L. Carry, Dianne Kinnon, Joan Killulark, Janet Nevala

**Affiliations:** 1 Consultancy for Alternative Education, Montreal; 2 Former senior program officer, Inuit Tuttarvingat, National Aboriginal Health Organization, Ottawa; 3 Former director, Inuit Tuttarvingat, National Aboriginal Health Organization, Ottawa; 4 Department of Health and Social Services, Nunavut; 5 Nevala Consulting, Ottawa

**Keywords:** Inuit, health workers, distance education, self-directed learning, tobacco reduction, Canada

## Abstract

**Background:**

Tobacco reduction is a major priority in Canadian Inuit communities. However, many Inuit frontline health workers lacked the knowledge, confidence and support to address the tobacco epidemic. Given vast distances, high costs of face-to-face training and previous successful pilots using distance education, this method was chosen for a national tobacco reduction course.

**Objective:**

To provide distance education about tobacco reduction to at least 25 frontline health workers from all Inuit regions of Canada.

**Design:**

Promising practices globally were assessed in a literature survey. The National Inuit Tobacco Task Group guided the project. Participants were selected from across Inuit Nunangat. They chose a focus from a “menu” of 6 course options, completed a pre-test to assess individual learning needs and chose which community project(s) to complete. Course materials were mailed, and trainers provided intensive, individualized support through telephone, fax and e-mail. The course ended with an open-book post-test. Follow-up support continued for several months post-training.

**Results:**

Of the 30 participants, 27 (90%) completed the course. The mean pre-test score was 72% (range: 38–98%). As the post-test was done using open books, everyone scored 100%, with a mean improvement of 28% (range: 2–62%).

**Conclusions:**

Although it was often challenging to contact participants through phone, a distance education approach was very practical in a northern context. Learning is more concrete when it happens in a real-life context. As long as adequate support is provided, we recommend individualized distance education to others working in circumpolar regions.

The purpose of this intervention was to use distance education with at least 25 frontline health workers from all Inuit regions of Canada (Inuit Nunangat) to help them reduce tobacco use in their communities. Health Canada funded this project.

## Context

There are 4 regions in Inuit Nunangat, from west to east: the Inuvialuit Settlement region with 6 communities; Nunavut with 26 communities; Nunavik with 14 communities; and Nunatsiavut with 6 communities. In almost all cases, these remote communities are not accessible by road. Population sizes according to 2011 census data vary from very small (from approximately 100 to 300), to medium (400 to 800), to large (up to 6 or 7,000 in the case of the largest regional/territorial capital). The vast majority of residents are Inuit/Inuvialuit.

In almost all communities, one or more Inuit/Inuvialuit lay health workers – overwhelmingly females – have been hired to work as community health workers. Some have received formal training; others have not. Most remain on the job for many years, assisting the nurse(s) in the clinic and doing health promotion work in the community. It was this category that we targeted for this intervention. To simplify, we refer to them as community health representatives (CHRs), even though they are known by different titles, such as community health workers, community wellness workers and so on. Many CHRs are themselves smokers, and as a result have been reluctant to do tobacco reduction work in their communities.

## Motivation: the need for an intervention

### Why tobacco reduction?

Tobacco reduction is of major concern to health practitioners and policy-makers globally (1–12). Given the higher rates of smoking in Aboriginal communities, tobacco reduction is of particular concern to people working in these communities (13–27). This applies equally to health workers in Inuit communities in Canada.

In the 1990s, 1 in 3 deaths in the former Northwest Territories (which includes what is now Nunavut) was attributable to smoking (28). Lung cancer rates for both Inuit women and men in Canada are the highest in the world (29), and a reduction in cancer rates would make the largest contribution to an increased life expectancy in Inuit Nunangat compared to the rest of Canada (30). Apart from cancer, tobacco use also contributes to increased rates of heart disease, stroke, respiratory illness and complications of pregnancy (22). Jenkins et al. (31) found that 85% of Inuit mothers in Iqaluit, Nunavut reported that they had smoked during pregnancy, and 94% of infants were exposed to tobacco smoke in the home. Smoking is the single greatest preventable contributor to death, disease and disability for Inuit. Despite this, smoking reduction is often neglected due to more immediate health crises or new funding priorities. This project aimed to keep tobacco reduction on the public health agenda in the communities from which participants were selected.

### Why distance education?

Access to post-secondary educational opportunities is very difficult for learners living in remote regions (32). Many CHRs are responsible for child- and/or elder-care in their families. It is much more practical for them to stay home rather than travel long distances (at high cost to the employer, in often unreliable weather conditions) to attend face-to-face workshops. In addition, as distance learners, they can stay on the job, taking a few hours off for telephone calls and project work related to their studies, instead of being absent for days or even weeks at a time, and having to be replaced at work. Also, studying by distance education enables participants to integrate their learning into their everyday work and undertake practical projects in the community, rather than being isolated in a workshop away from the reality of the community health problems they wish to address.

There are, however, several barriers to be overcome in distance education programmes, one of which is high dropout rates. Hamilton et al. (33) had “active participation” from only 46%, “less active participation” from 24%, and non-participation from 30% of the 96 Inuit students who registered for an on-line nutrition course. (Note: The target students for their course were educationally, occupationally, socially and culturally very similar to those in this intervention.) Language and cultural barriers for Inuit students studying in English, work pressures and geographic isolation of students were other issues to be considered in both this case and the Hamilton study. Using distance education that includes intensive, on-going support with Inuit adult learners has worked very well in a similar program where 81% of participants completed the course, thus confirming the potential of culturally affirming and learner-supportive distance education in Inuit Nunangat (34).

### Why a self-directed approach to learning?

The trainers (RC & MH) had had positive prior experience using self-directed learning with distance learners in a course for primary health-care educators in South Africa (35). They therefore recommended this approach for the current intervention.

Self-directed learning addresses differences in previous training, levels of education and experience, and variations in working conditions of frontline health workers. Learners identify their own learning needs and customize their learning to meet those needs. There is a flexible timetable and structure to accommodate the varied circumstances of participants from different regions across Canada.

### Review of tobacco reduction literature and resources

In 2010, Inuit Tuttarvingat of the National Aboriginal Health Organization contracted RC & MH to conduct a review of relevant literature, resources and promising practices for the Inuit Tobacco-free Network, a national group working to reduce tobacco use in Inuit communities. The report (36) ensured that preparations for the planned distance education course in tobacco reduction were fully informed by practices of possible relevance to Inuit – practices that were found to be promising with various populations (with a focus on Aboriginal communities) in Canada and globally. The review confirmed that many promising practices in tobacco reduction were already being implemented in Inuit regions. Successful programmes tend to have a community rather than individual orientation, use materials and approaches that have a high degree of relatedness to the culture and community, and continue over several years.

A summary of the comprehensive literature review, titled *What Works in Reducing Tobacco Use in Indigenous Communities? A Summary of Promising Practices for Inuit* (37), was produced in 3 dialects of the Inuit language, English and French, and this was distributed to course participants, partners and other stakeholders. The comprehensive report (36) was web-published after the course.

## The distance education course

### The National Inuit Tobacco Task Group (Advisory Group)

The Advisory Group consisting of stakeholders, elders and experts from across Inuit Nunangat guided this project. Up to 10 members simultaneously joined teleconference calls to give feedback on draft materials, and 20 local supervisors, usually the nurse-in-charge, and 5 managers from regional/territorial health departments also assisted.

### Trainers

Two experienced trainers (non-Inuit, but who had both worked in Inuit communities for many years) helped design and implement the distance education course and provided follow-up support after the project ended.

### Recruitment of participants

**Fig. 1:**
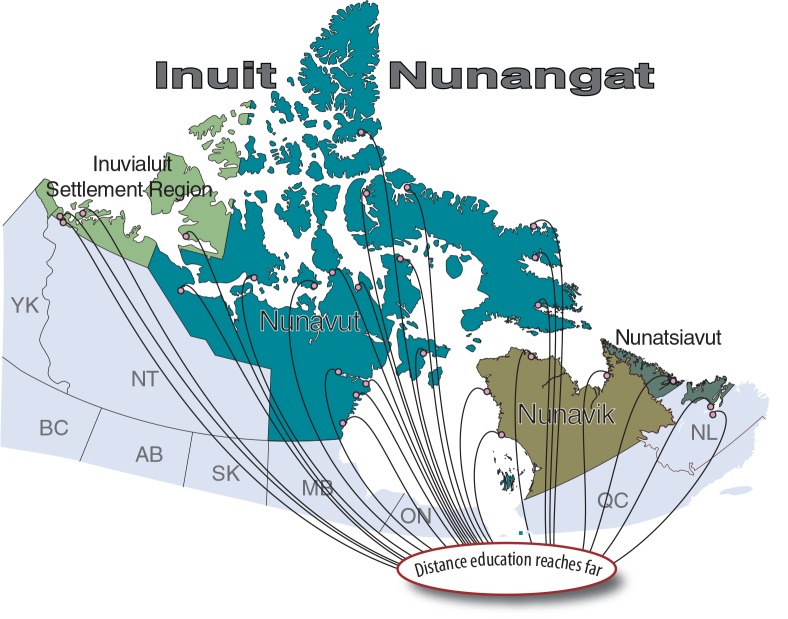
Twenty-eight participants participated from all regions of Inuit Nunangat; the trainers were based in Montreal.

Inuit regional and territorial health authorities, non-governmental organizations, hamlet councils, health centres and CHRs in almost every Inuit and Inuvialuit community were contacted to support the project and to help recruit participants. Telephone and/or e-mail contact was established with everyone on a list of 33 potential recruits. Three people declined to participate, and two more dropped out very early on. As shown in Fig. 1, that left 28 frontline health workers from all 4 regions of Inuit Nunangat actively participating in the course. There was an excellent spread of participants from the 4 Inuit regions, with greatest participation from the Inuvialuit Settlement region (4 participants from 6 communities, or a 67% participation rate) and Nunavut (17 participants from 26 communities, or 65%). Nunatsiavut had 3 participants from 6 communities (50%), and Nunavik had 4 participants from 14 communities (29%).

There was a good balance of participants from small, medium and large communities – something the Advisory Group had specifically requested. Some participants had previous training in tobacco reduction; others did not. The majority were CHRs. With one exception, all participants were Inuit/Inuvialuit (96%), and all but 2 were women (93%).

### Dates

The course ran from 1 March to 31 May 2010. On-going support was then provided to participants as needed after the official end of the course.

### Educational approach

The programme used “asynchronous” distance education: participants worked on their own schedule and had one-on-one contact with the trainers at a time that suited them and their work schedules. (In “synchronous” learning, on the other hand, all students are present at the same time, for example, for a videoconference or conference call.) We used individual telephone calls, mail, e-mail and fax to communicate. E-mail access is unfortunately still very limited for many frontline health workers in Inuit Nunangat. Many share an office, and some have never been adequately trained in the use of computers. Telephone was therefore the communication method of choice, but it sometimes took several attempts as participants were often out in the community or assisting the nurse in the health centre when we called.

This distance education course included elements of self-directed learning. We recognized that each person had unique past experiences and learning needs, so we wanted to offer a course that would:

- recognize their prior knowledge and experience;
- involve them in identifying their own learning needs and planning how best to meet them – directing and managing their own learning (with the trainers’ help);
- support them with individual telephone calls (and e-mail where appropriate), and continue to provide follow-up support after the course ended if desired;
- enable them to integrate their learning into their everyday work and help them to implement practical projects in their communities; and
- build their capacities as health workers so that they could better help to improve the health of their communities.

A self-directed course like this places the learner – not the trainer – at the centre. And because each participant is different, a pre-packaged approach is much less effective than a self-directed approach. We have found over many years that this is the best way to work with adults as they continue to develop their knowledge, attitudes and skills for their jobs.

### Course options, learning materials and core competencies

**Fig. 2:**
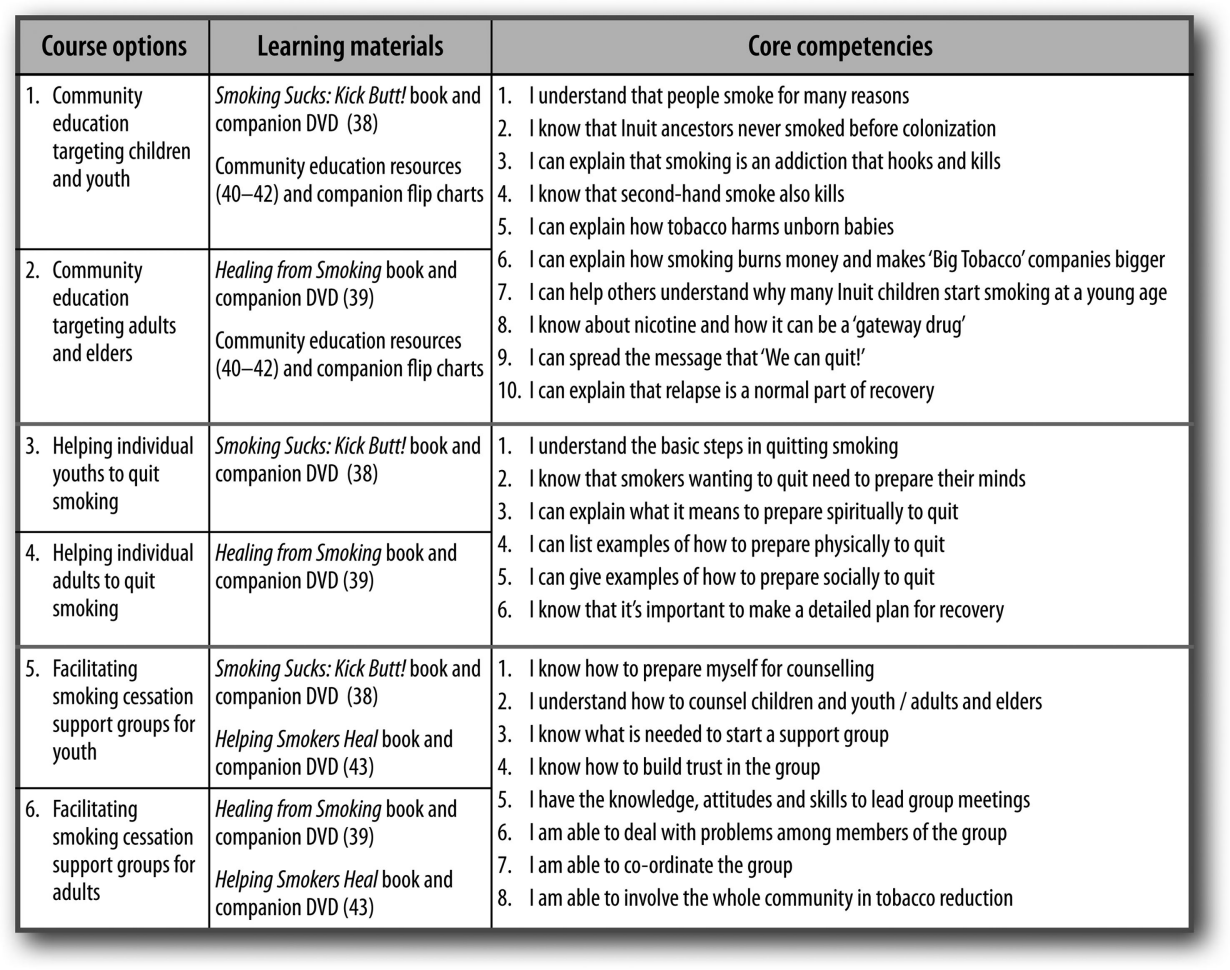
Course options, learning materials and core competencies.

Participants selected their main area of interest from a “menu” of 6 course options. Each option required the study of different learning materials, and core competencies were specified for each option (see Fig. 2).

### Pre- and post-test self-assessments

Once participants had chosen from the menu of 6 course options, they completed the relevant section of a pre-test self-assessment tool that contained a total of 24 core competencies (specified in Fig. 2) and 176 sub-competencies. (Every core competency was divided into between 2 and 4 detailed sub-competencies, for a total of 176.) Each participant only had to complete the relevant section of this self-assessment, depending on which course option he or she chose.

The self-assessment form included 2 colour-coded columns, the first for the pre-test designed to assess learning needs and the second for the post-test. Participants used a four-point rating scale: 1 meant “I know nothing about this”; 2 meant “I need to learn a lot about this”; 3 meant “I only need to learn a bit about this”; and 4 meant “I don't need to learn any more about this”. By rating themselves as mentioned earlier before and after the course, they were able to “measure their learning” on each detailed sub-competency.

### Community projects

Participants selected a community project to implement (choosing 1 of 12 options provided, or designing a unique project in consultation with their trainers), and used the course-related books, DVDs, flipcharts and telephone calls with trainers to prepare themselves for their projects.

Examples of suggested projects were:

- Prepare a talk to give at the school about the health effects of tobacco use (what teaching methods and aids would you use?);
- Learn about withdrawal symptoms and how to explain them to smokers;
- Host a phone-in radio show about tobacco reduction;
- Design your own project and negotiate with your trainer to do that instead of one of the suggested projects.

### Flexibility

The duration of the course (between 20–40 hours over 3 months) was flexible to take into account the differences in projects and seasonal activities across the regions. Other issues such as vacation or sick leave, competing study programs, shortages of clinic staff and the pattern of life in each community all had a bearing on the time needed for each participant to complete their course. Flexible learning opportunities are particularly important for Aboriginal learners (32,44,45). Studies were completed in on-duty time.

### Communication and support during and after the course

Personal contact with participants was through phone, e-mail and fax. The main incentive for remaining in this program was the personal support participants had from trainers and the new tobacco education resources they were trained to use and had available for their work in the community. Each participant followed their own customized programme and had regular contact through telephone with the trainers.

After the course had officially ended, the trainers offered follow-up support to participants to undertake activities to continue addressing tobacco reduction in their communities.

**Fig. 3:**
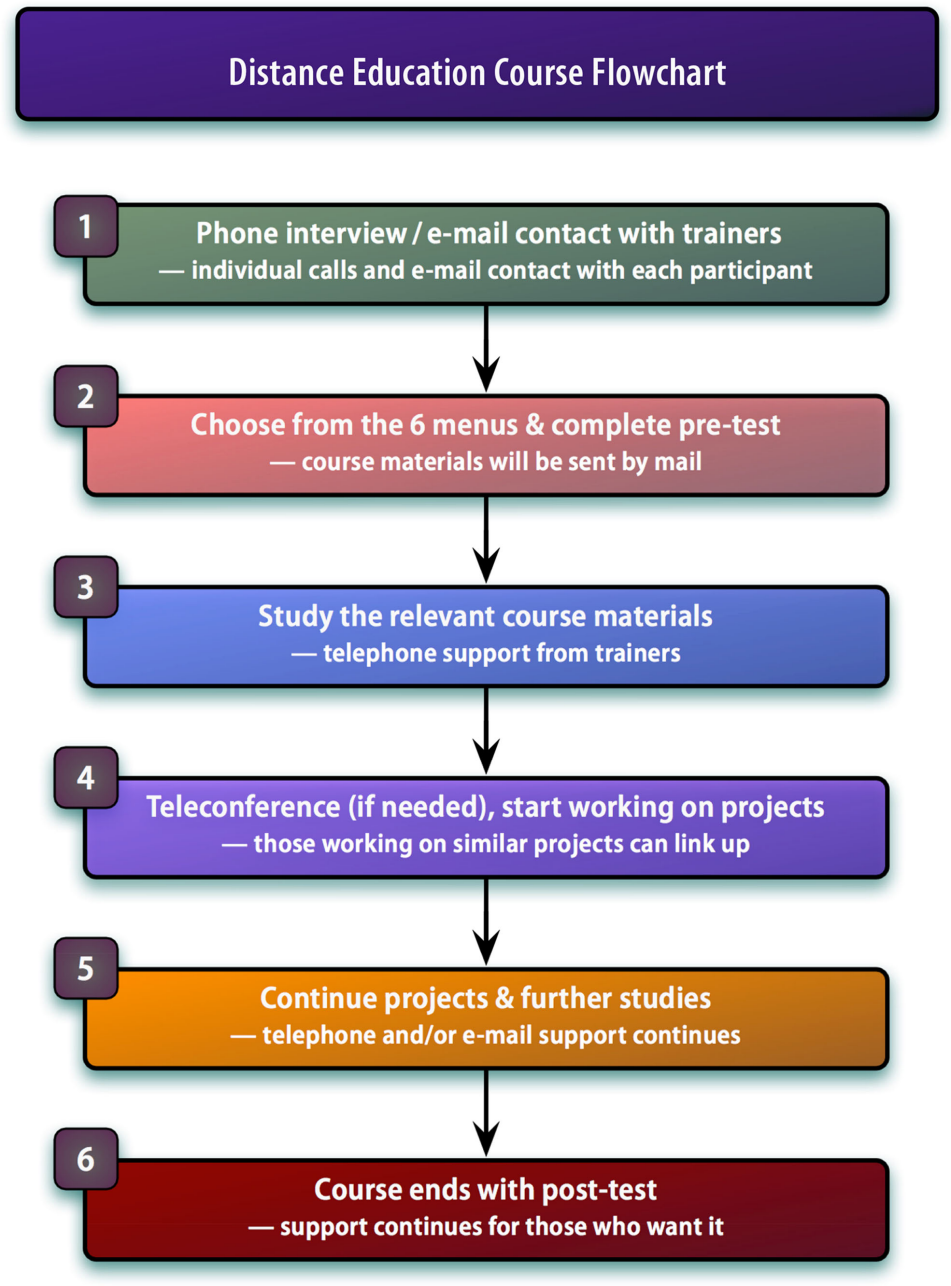
The 6-step course flowchart.

To summarize, Fig. 3 shows a flowchart with the 6 main steps used in the course.

## Results and discussion

### Course completion

The criteria for course completion were: (a) participants completed regular phone calls with the trainers in which they showed progress on studying the learning materials provided; (b) completed the pre- and post-tests; and (c) completed an approved community project to the satisfaction of the trainers.

Of the 30 original participants, 27 (90%) completed the course. (If we exclude the 2 people who dropped out right at the outset, the completion rate is 27 out of 28, or 96%.)

### Pre- and post-tests

The mean score for pre-tests was 72% (range: 38–98%). To help consolidate learning and fill in gaps, participants used the post-test tool with an open book. This necessarily produced a 100% score because, where needed, they were directed to the correct answer with page references to complete any incomplete or incorrect answers. While this obviously cannot qualify as a true post-test, the mean improvement over pre-test scores was 28% (range: 2–62%).

### Phone calls

Calls lasted from 10 to 45 minutes each. These calls were essentially individualized telephone tutorials. More than 420 calls were made during the project.

### Community projects

Participants completed some excellent community projects as part of the course. Examples include:

- Offered individual counselling to colleagues at the clinic who wished to quit but kept relapsing;
- Offered a support group for smokers who wished to quit;
- Ran educational sessions in the school using the *Smoking Sucks* book and DVD (several participants chose this option, and some invited youth to co-facilitate sessions with them);
- Launched a poster competition about the harmful effects of smoking;
- Worked on own personal plan to quit smoking using the resources from the course and with encouragement from family.

### Feedback from participants

At the end of the course, when the trainers telephoned each participant to do the post-tests, they asked for feedback about various aspects of the course. Comments were typed as people spoke. (Note that efforts to reach the supervisors of course participants for their feedback were unsuccessful due to high staff turnover and annual and other leaves causing temporary replacements to be in charge when we tried to contact people.)

#### Learning materials

Participants commented that they appreciated the supplied learning materials for their accessibility, cultural appropriateness, Inuit content and Inuktitut syllabic translation. They mentioned that before the course they had had few if any new, appropriate materials for their community education work, or to give to clients.

- It's about time that we got some new resources for tobacco education. My old [resources] have been seen many times before so now with the new books, [people] will be interested.
- I read the books and they are really good. I'll use them every time now and they'll really help the smokers I am working with … Now with *Healing from Smoking and Helping Smokers Heal* I will have the help that I didn't have before.
- I really like *Healing from Smoking* and particularly the illustrations – they make it easy for people to understand and to want to learn more. My next step will be to facilitate a study group of the health workers at the clinic who are smokers [using the book] …
- People who look into those books of yours *[Smoking Sucks: Kick Butt!]* are all wanting to have a copy for themselves, so I just gave them all out and now I need more.
- I have 3 smokers at home even while I am a non-smoker, so I get frustrated – I don't know how to talk to them. Now I feel more like trying again – with help from *Healing from Smoking and Smoking Sucks*.

#### Telephone support

As a result of the on-going support during the course, the trainers informed and inspired participants to keep trying to reduce tobacco use in their communities.

- Having an update like this course really helps to get me focused on tobacco again because there are so many other crises coming up.
- This talk [phone conversation with trainer] is helping me to get my confidence again. I lost confidence in myself and felt guilty when I started smoking again. That was after 10 years of being a non-smoker.
- I feel bad about doing nothing on tobacco for more than two years now, so I am pleased to get some help to start again.

#### Quitting smoking

A key part of the philosophy of this course was that even current smokers can encourage others to quit or not to start smoking. We never “blamed or shamed” CHRs who were themselves smokers. Nevertheless, several participants used this opportunity to quit smoking.

- I quit smoking in April, shortly after the course started, and have not smoked since. A group of 8 women decided to quit to support me, and after several months, only one has relapsed.
- I surprised myself by quitting smoking on 10th March. I'd never tried to quit before. What helped was that I wanted to be able to help others. The patch was helpful. Before, I even used to get up in the middle of the night for a cig. Friday, 30 July 2010: I am still a non-smoker and am incredibly proud and surprised at myself for quitting … I am just so happy that I got the opportunity to do this course.

### Follow-up support

Follow-up support often made the difference between a trainee staying active in tobacco reduction and simply going back to the *status quo* before training, where this topic is neglected due to more immediately pressing health problems in the community.

Examples of follow-up support that made a difference include one CHR who used what she had learned during the course to be a co-trainer in a Government of Nunavut-sponsored *Smoking Sucks* workshop for Inuit youth from the Kitikmeot region, and to be a mentor to the youth who attended the workshop during their subsequent community projects. (The lead trainer on the *Smoking Sucks* workshop was one of the trainers from this distance education course.) She stated that in the period before her course began, she had stopped doing tobacco education because there was not much community support. However, the course and the follow-up engagement with the project team re-kindled her confidence and enthusiasm to work on tobacco reduction again.

Another CHR was also a co-trainer in a *Smoking Sucks* workshop in the Kivalliq region of Nunavut. Her studies on the distance education course put her in a good position to be a co-trainer, also working together with one of her trainers from this distance education course.

In 2 other communities, course participants were working on a 6-year community-based participatory research project called ‘Changing the culture of smoking’. Examples of projects they have worked on in that capacity include offering annual community-wide smoking cessation challenges, doing a smoke-free homes survey, running tobacco education sessions in the schools and organizing community events such as radio shows and community quizzes to raise awareness about tobacco use. In all cases, participants reported that their learning on the distance education course has been very helpful.

## Conclusion

The very high completion rate (90%) achieved for this course shows that there is a clear role for individualized distance education that is flexible, culturally affirming, and learner-supportive. Face-to-face workshops in Inuit regions are extremely difficult to organize due to vast distances, high travel and accommodation costs and extreme weather which may prevent participants from attending. As long as adequate and on-going support is provided, we recommend this approach to others working in circumpolar regions.

Competency-based self-directed distance education is not simply a cheap alternative to face-to-face training. Its real strength is that it goes much further, much “deeper” – by consolidating “learning and doing” in the learner's real-life work setting. The one-on-one approach accommodated varying levels of past experience and different learning needs and styles, while following up with and supporting each participant. With relevant resources and the opportunity to practise using them while receiving support from trainers, participants provided improved access to tobacco cessation and prevention services for their communities.

The trainers felt that this intensive approach would have been better done with a smaller group: supervising 28 individualized programs was a real challenge, especially given the limited use of e-mail. Also, a multi-year approach would consolidate learning and improve outcomes in successive community interventions, allowing participants to build on knowledge and skills gained earlier, and take their tobacco reduction practice further. Inoculation against the epidemic of tobacco use cannot be accomplished with a single jab.
